# Towards Streamlined Transparent Data Linkage

**DOI:** 10.23889/ijpds.v9i5.2577

**Published:** 2024-09-18

**Authors:** Amrita Bandyopadhyay, Sinead Brophy, Natasha Kennedy, Hope Jones, Julie Evans, Mark A Bellis, Benjamin Rowe, Irena Spasic, Cynthia L. Mcnerney, Simon Moore

**Affiliations:** 1 National Centre for Population Health and Wellbeing Research, Swansea University Medical School; 2 Heath Data Research UK; 3 Administrative Data Research Wales, Swansea University Medical School; 4 Public Health Wales; 5 Liverpool John Moores University; 6 South Wales Police; 7 School of Computer Science & Informatics, Cardiff University; 8 SAIL Databank, Swansea University Medical School; 9 Security, Crime & Intelligence Innovation Institute and Violence Research Group, School of Dentistry, Cardiff University

## Introduction

The UK Government increasingly emphasises a comprehensive, multi-agency approach to tackling crime. This pilot study explores the feasibility of integrating police data with routine health data to develop a holistic understanding of the predictors of domestic abuse (DA).

## Approach

The study encompasses three work-packages a) coding the narrative data from Domestic Abuse, Stalking and Harassment (DASH) risk assessment report from Public Protection Notifications (PPNs), which are information-sharing documents recording safeguarding concerns and shared with partner agencies b) exploring to identify what works to harmonise different software systems within police data-sharing approach, c) constructing a case study by linking PPN DASH data with routine health and administrative records. These efforts illustrate the potential of national data-sharing initiatives.

## Results

a) Text mining methods successfully identified and coded (with over 95% accuracy) 17 refereed/involved agencies from the PPN DASH data.

b) Barriers to data sharing were attributed to a lack of clarity and consensus regarding appropriate information sharing, rather than technical obstacles. This barrier can be overcome by an unambiguous framework, endorsed at an elevated level, highlighting which data should be shared.

c) The data-linkage study revealed that victims of DA had prior interactions with healthcare services before their initial PPN, and younger pregnant victims had higher risk of future healthcare emergency visits.

## Conclusion and Implication

Police and health data integration enhances evidence-based prevention and early identification of vulnerable individuals by both law enforcement and public health services.

